# The Physician’s Pocket Day-Book

**Published:** 1892-12

**Authors:** 


THE PHYSICIANS' POCKET DAY-BOOK.—Designed by C. Henri Leonard, M. A., M. D., Detroit, Mich. Price, $1.00. Issued annually by the Illustrated Medical Journal Co., Detroit, Mich.

ACCOMMODATES daily charges for twenty-five or fifty families weekly, has complete obstetrical record for ninety-four cases and monthly memoranda for debit and credit cash account. [illegible] s t of doses of old and new drugs, number of drops in twenty minims of the chief fluid medicaments, poisons and their antidotes, tests for urinary deposits, exanthematicæ, obstetric calendar, disinfectants, weights, measures, etc. This little book is well bound and altogether well adapted to the purpose for which it is intended. C. E. J.

